# Process evaluation of a cluster-randomised trial testing a pressure ulcer prevention care bundle: a mixed-methods study

**DOI:** 10.1186/s13012-017-0547-2

**Published:** 2017-02-13

**Authors:** Shelley Roberts, Elizabeth McInnes, Tracey Bucknall, Marianne Wallis, Merrilyn Banks, Wendy Chaboyer

**Affiliations:** 1 National Centre of Research Excellence in Nursing, Menzies Health Institute Queensland, Griffith University, Gold Coast Campus, Southport, QLD 4222 Australia; 2 Nursing Research Institute, St Vincent’s Health Australia (Sydney) and School of Nursing, Midwifery and Paramedicine, Australian Catholic University, North Sydney, NSW 2060 Australia; 3 Centre for Quality and Patient Safety, School of Nursing and Midwifery, Deakin University, Geelong, VIC 3220 Australia; 4 Alfred Health, Melbourne, VIC 3004 Australia; 5 School of Nursing, Midwifery and Paramedicine, University of the Sunshine Coast, Locked Bag 4, Maroochydore, QLD 4558 Australia; 6 Department of Nutrition and Dietetics, Royal Brisbane and Women’s Hospital, Herston, QLD 4019 Australia

**Keywords:** Care bundle, Cluster-randomised trial, Complex intervention, Patient participation, Pressure injury prevention, Pressure ulcer prevention, Process evaluation

## Abstract

**Background:**

As pressure ulcers contribute to significant patient burden and increased health care costs, their prevention is a clinical priority. Our team developed and tested a complex intervention, a pressure ulcer prevention care bundle promoting patient participation in care, in a cluster-randomised trial. The UK Medical Research Council recommends process evaluation of complex interventions to provide insight into why they work or fail and how they might be improved. This study aimed to evaluate processes underpinning implementation of the intervention and explore end-users’ perceptions of it, in order to give a deeper understanding of its effects.

**Methods:**

A pre-specified, mixed-methods process evaluation was conducted as an adjunct to the main trial, guided by a framework for process evaluation of cluster-randomised trials. Data was collected across eight Australian hospitals but mainly focused on the four intervention hospitals. Quantitative and qualitative data were collected across the evaluation domains: recruitment, reach, intervention delivery and response to intervention, at both cluster and individual patient level. Quantitative data were analysed using descriptive and inferential statistics. Qualitative data were analysed using thematic analysis.

**Results:**

In the context of the main trial, which found a 42% reduction in risk of pressure ulcer with the intervention that was not significant after adjusting for clustering and covariates, this process evaluation provides important insights. Recruitment and reach among clusters and individuals was high, indicating that patients, nurses and hospitals are willing to engage with a pressure ulcer prevention care bundle. Of 799 intervention patients in the trial, 96.7% received the intervention, which took under 10 min to deliver. Patients and nurses accepted the care bundle, recognising benefits to it and describing how it enabled participation in pressure ulcer prevention (PUP) care.

**Conclusions:**

This process evaluation found no major failures relating to implementation of the intervention. The care bundle was found to be easy to understand and deliver, and it reached a large proportion of the target population and was found to be acceptable to patients and nurses; therefore, it may be an effective way of engaging patients in their pressure ulcer prevention care and promoting evidence-based practise.

## Background

Pressure ulcer (PU) affects around 10–30% of hospitalised patients worldwide [1–4] and contributes to significant patient burden [5, 6] and health care costs [7]. PUs increase hospital length of stay [8] and treatment costs across Australian public hospitals were estimated at A$983 million per year, representing 1.9% of all public hospital expenditure in 2012–2013 [9]. The majority of PUs in the clinical setting are hospital-acquired pressure ulcers (HAPU) [10–12], most of which are preventable [13–15]. As such, many health care organisations around the world have implemented financial penalties such as withdrawal of reimbursement or fines to hospitals for HAPU [16–18]. Pressure ulcer prevention (PUP) is also one of the national health service standards set by the Australian Commission on Safety and Quality in Health Care [13], highlighting the importance of this clinical issue.

Evidence suggests that patient outcomes can be improved and adverse events reduced through the use of multi-component interventions, or care bundles [19–22] and patient participation in care [23]. Therefore, an innovative PUP care bundle (PUPCB) was developed by our research team to reduce HAPU in patients at high risk of developing PU [24]. The INTroducing A Care bundle To prevent pressure ulcer (INTACT) trial was a pragmatic cluster-randomised trial (c-RT) assessing the effect of a PUPCB on the development of PU in hospitalised patients deemed at risk of PU (i.e. with reduced mobility) [25]. Eight Australian hospitals were randomised to receive the intervention or control. The intervention was aimed at both the cluster (nurse) and individual (patient) level, focusing on patient participation in care and partnerships between patients and nurses in PUP. The PUPCB was delivered to patients by a trained research assistant and included a brochure, poster and DVD, all of which contained three key messages: (1) keep moving; (2) look after your skin; and (3) eat a healthy diet. These messages did not vary between different patient groups and were based on current literature and international evidence-based guidelines, which indicate that immobility, poor skin hydration and malnutrition are modifiable risk factors for PU [26–29]. If patients’ relatives were present at the time of intervention delivery, they would have also received the information; however, they were not specifically targeted. The cluster-level intervention involved information sessions delivered to nursing staff on study wards, who were given education and training around PUP, the PUPCB, and partnering with patients in PUP care. Further detail on the theory underpinning the intervention and study methods are reported elsewhere [24, 25].

Care bundles are multifaceted, containing a number of interacting components; they depend on the social context and behaviours of those delivering or receiving the intervention and may target a number of groups or organisational levels [30, 31]. Due to these complexities, such interventions pose methodological challenges, and study outcomes (i.e. intervention success or failure) may be affected by factors related to the implementation or delivery of an intervention [30]. Therefore, evaluation of the processes related to intervention delivery is important to provide insight into why interventions work or fail and how they can be improved [30]. Process evaluation is especially important in multisite trials where the same intervention may be implemented and received in different ways [31].

The aim of this study was to evaluate the processes underpinning implementation of the PUPCB and patient and staff perceptions of the bundle in order to give a deeper understanding of the effects of the intervention.

## Methods

### Study design

A pre-specified mixed-methods process evaluation was conducted as an adjunct to the INTACT study, guided by a framework for c-RTs described by Grant et al. [32]. A number of aspects comprised the process evaluation, as described in Table 1. Recruitment and reach, intervention delivery, acceptability of the intervention and potential for maintenance of the intervention (i.e. in standard care) were all measured at the cluster and individual levels, using a mix of quantitative and qualitative approaches. These were interpreted with consideration of context and in relation to the main trial’s findings.

**Table 1:** Components and methods of process evaluation, adapted from Grant et al. [31]

Evaluation domain	Research questions	Research methods	Data collection tools
Recruitment of clusters	How are clusters sampled and recruited? Are they representative?	Descriptive analysis of recruited clusters and their representativeness	Hospital demographics were collected by CIs at each site
Recruitment and reach in individuals	Who actually receives the intervention in each setting? Are they representative?	Descriptive analysis of screening log data	Recruiting RAs at each site recorded total number of patients screened vs. total number of patients recruited and barriers to participation, reasons for dropout
Delivery to clusters	What intervention is actually delivered to each cluster? Is it the one intended by researchers?	Descriptive analysis of intervention delivery (i.e. training sessions) to nursing staff at each site	CIs kept a log of information sessions delivered to nursing staff at each site, including number of participants and time spent at each session, who delivered the session and when (in relation to study timeline)
Delivery to individuals	What intervention is actually delivered to individuals? Is it the one intended by researchers?	Descriptive analysis of intervention delivery (i.e. DVD, brochure, poster)	Interventionist RAs recorded which intervention components were delivered to each patient and total time spent with each patient
Response of clusters	How is the intervention adopted by clusters?	Qualitative analysis of nurse interviews	A semi-structured interview guide was used to explore nurses’ perspectives of the intervention (four to five nurses per intervention site) (citation masked for peer review)
Response of individuals	How does the target population respond?	Qualitative analysis of patient interviews	A semi-structured interview guide was used to explore patients’ perspectives of the intervention (four to five patients per intervention site) (citation masked for peer review)
Maintenance	How and why are these processes sustained over time (or not)?	Qualitative analysis of nurse and patient interviews	See above (for nurses and patients)
Effectiveness	What are the effects on primary and secondary outcomes?	Exploratory analyses of effect of trial processes (i.e. above domains) on trial outcomes	Main findings from the INTACT trial and results from above process evaluation domains comprised data for these analyses. However, the study was not powered for these exploratory analyses
Unintended consequences	Are there unintended changes in processes and outcomes, both related to the trial intervention and unrelated care?	Qualitative analysis of observed data, qualitative analysis of patient and staff interviews	Semi-structured interview guides and observational tools/logs (described above)
Context	What is the wider context in which the trial is being conducted?	Consideration of state and national contexts of each hospital setting regarding PUP (i.e. national standards, state penalties, hospital policies and procedures) when interpreting findings	Literature search of local, state and national hospital standards, policies and procedures relating to PUP
Theory	What theory has been used to develop the intervention? Can a theory be considered to interpret the effects of the intervention?	N/A–consideration of theory used to develop the intervention was considered in interpretation of findings. Additional theories were sought to explain findings of each component of the process evaluation	

*CIs* chief investigators, *RAs* research assistants

### Setting and sample

Eight Australian hospitals participated in the INTACT trial, and data for the process evaluation was collected across all sites, but focused mainly on the four intervention sites. Participants included patients and nurses on study wards at intervention sites, but some data (such as recruitment data) was also collected on patients at control sites. For qualitative data collection, patients and nurses provided individual informed consent to participate in interviews.

### Data collection

Quantitative data were collected mostly prior to and during the intervention, whilst qualitative data were collected post-intervention. Equal emphasis was given to quantitative and qualitative data, which complemented each other in terms of interpreting findings. The research questions, research methods and data collection techniques for each evaluation domain are described in Table 1.

### Data analysis

#### Recruitment and reach

The representativeness of clusters was assessed qualitatively by comparing hospital type, location, services and size to all Australian hospitals using Australian Institute of Health and Welfare statistics. Recruitment of individuals was assessed by comparing the total numbers of patients screened and recruited at each site to determine the proportion of the target population included in the study. This evaluation domain was assessed to provide insight into the sample and its representativeness.

#### Intervention delivery

Data on intervention delivery to clusters were analysed descriptively (i.e. number of sessions delivered, number of staff present at each session, time spent delivering each session, who delivered it and when it was delivered in relation to the study timeline). Intervention delivery to patients (i.e. components of the PUPCB delivered and time spent with patients) were analysed descriptively. Chi-squared tests and one-way analysis of variance (ANOVA) tests were used to determine any differences in intervention delivery between sites. The effects of intervention dose on main trial findings were also analysed at the individual patient level using chi-squared tests (i.e. number of intervention components delivered) and one-way ANOVA tests (i.e. time spent delivering intervention) to describe any associations between intervention delivery and PU development.

#### Response to intervention

Semi-structured interview data on responses of clusters (i.e. nurses) and individuals (i.e. patients) to the intervention were analysed qualitatively using thematic analysis to give in-depth insights into patients’ and nurses’ perceptions of the intervention. More detailed methods of patient and staff interviews are reported elsewhere (citations masked for peer review).

## Results

The outcomes of the INTACT trial are reported elsewhere (citation masked for peer review); however, for the purpose of this process evaluation, a summary of the main trial findings are presented. PU occurred in 6.1% of intervention and 10.5% of control patients, and PU incidence was 9.6 and 20.1 per 1000 person-days in the intervention and control groups, respectively (incidence rate ratio 0.48; 95% CI 0.33, 0.69; *p* < 0.001). After adjusting for clustering and covariates (age, baseline PU, body mass index, admission cause, residence and comorbidities), the hazard ratio was 0.58 (42% risk reduction, *p* = 0.198). However, the intra-class correlation coefficient was higher than expected (i.e. higher than that used to calculate sample size) so it is likely the study was underpowered. There are other possible reasons for these results, such as potential baseline differences, or the delivery of the intervention which is the focus of this evaluation.

### Recruitment of clusters

Clusters were recruited using a convenience sample of hospitals meeting the study’s inclusion criteria, detailed elsewhere (citation masked for peer review). The study included eight hospitals representing health services across three states in Australia. Six public (75%) and two private (25%) hospitals were included in the study, which is similar to the total split of public (72%) and private (28%) hospitals across Australia [33]. Hospitals were metropolitan (*n* = 7) or regional (*n* = 1) and were either tertiary (*n* = 5), quaternary (*n* = 2) or tertiary, providing quaternary services (*n* = 1), reflecting the range in Australian hospitals. The size of study sites ranged from 270 to 929 beds (median 565, IQR 355–737 beds) and 8–28 wards (median 18, IQR 10–23 wards). Between four and 12 medical, surgical and rehabilitation wards (median 7, IQR 4–9 wards) were included in the INTACT trial across study sites.

### Recruitment and reach in individuals

The target population was patients who met eligibility criteria for inclusion in the study. The proportion of the total target population that was included in the study was 67.2%. Table 2 shows the total number of eligible patients and the numbers and proportion of refusing, consenting and included patients in the INTACT trial, which were similar between groups.

**Table 2:** Recruitment of patients

Enrolment procedure	Intervention *n* (%)	Control *n* (%)	Total *n* (%)
Eligible	1209	1168	2377
Refused	409 (33.8)	368 (31.5)	777 (32.7)
Consented	800 (66.2)	800 (68.5)	1600 (67.3)
Included^a^	799 (66.1)	799 (68.4)	1598 (67.2)

^a^One patient in each group was excluded after giving consent, as they met exclusion criteria (i.e. were confused)

### Intervention delivery to clusters

At the cluster level, the intervention was delivered to nurses on study wards. All four intervention sites held formal information sessions for nurses using a standardised PowerPoint presentation and study flyers, containing information on the intervention and partnering with patients in PUP. Between four and eight, formal sessions were conducted at each site (depending on the number of study wards at that site) prior to and during data collection, reaching 38–66 participants at each site. Formal inservices lasted 15–30 min and were delivered by the site Chief Investigator or a research assistant at each study site. In addition, all sites conducted informal sessions with nurses on study wards, which included an overview of the information presented in formal sessions. These informal sessions were conducted ad hoc, at times of convenience to ward staff, and may have been delivered to staff one-on-one or in groups. Due to the ad hoc nature of these informal sessions, it was difficult to quantify the number of nurses reached, but there were at least 30 informal sessions delivered at each site. These tended to be shorter than the formal presentations (around 3–10 min). While all clusters received a similar dose of the intervention in terms of content, frequency and time, individual nurses may have been missed and it was a one-off training session without ongoing support.

### Intervention delivery to individuals

Of the 799 intervention patients included in the study, 773 (96.7%) received at least one component of the intervention while 690 (86.4%) received all three components (i.e. brochure, poster and DVD). Table 3 shows the number of patients who received each component of the intervention across all sites. There were no significant differences in the proportion of patients who received each intervention component between sites.

**Table 3:** Intervention delivery to individuals

Site	Brochure *n* (%)	Poster *n* (%)	DVD *n* (%)	All components *n* (%)
1	193 (97.0)	185 (93.0)	174 (87.4)	167 (83.9)
2	193 (96.5)	187 (93.5)	180 (90.0)	176 (88.0)
3	192 (96.0)	192 (96.0)	171 (85.5)	171 (85.5)
4	192 (96.4)	194 (97.0)	180 (90.0)	176 (88.0)
Total	770 (96.4)	758 (94.9)	705 (88.2)	690 (86.4)

Of the 26 participants that did not receive the intervention after consent, reasons included the patient being discharged (*n* = 8) or too sick (*n* = 1), or the patient refused (*n* = 5) or withdrew from the study (*n* = 4), or data was missing (*n* = 3) or listed as ‘other’ reason (*n* = 5).

The mean (±SD) time spent delivering the intervention to each patient was 9.5 ± 5.4 min (range 0–45 min, median 8.0, IQR 7.0–11.0 min). There was a significant difference in time spent on intervention delivery between sites, using a one-way ANOVA (*p* < 0.001) and Tukey post hoc test (*p* < 0.001), as shown in Table 4. The amount of time spent delivering the intervention was also related to the number of components delivered (see Table 4). Interestingly, the most time was spent when patients only received one resource.

**Table 4:** Time spent delivering intervention to patients

Site	Time (min) mean ± SD	Number of components received	Time (min) mean ± SD
1	11.0 ± 6.7*	0	0.1 ± 0.4***
2	13.3 ± 5.3*	1	15.7 ± 25.4****
3	6.6 ± 2.5**	2	5.3 ± 5.6***
4	7.2 ± 2.4**	3	10.3 ± 4.6****
Total	9.5 ± 5.4		

*Different from all other sites (*p* < 0.05)

**Different from sites 1 and 2 (*p* < 0.05)

***Different from all other numbers of components received (*p* < 0.05)

****Different from 0 to 2 components received (*p* < 0.05)

### Response of clusters

A total of 18 nursing staff participated in interviews across the four intervention sites. Nurses’ perceptions of the intervention were expressed in five categories: (1) awareness of the pressure ulcer prevention care bundle and its similarity to current practise; (2) improving awareness, communication and participation with the pressure ulcer prevention care bundle; (3) appreciating the positive aspects of patient participation in care; (4) perceived barriers to engaging patients in the pressure ulcer prevention care bundle; and (5) partnering with nursing staff to facilitate pressure ulcer prevention care bundle implementation. Nurses responded well to the PUPCB when they thought it reflected or supported their current PUP practise. They realised benefits to both the PUPCB and patient participation in general, and this increased their acceptance for both. Nurses also provided insights into implementation and sustainment of such an intervention in practise; they spoke about communication and dissemination of results, the importance of leadership and influence and keeping the bundle simple to deliver. A detailed analysis of clusters’ response to the intervention is reported elsewhere (citation masked for peer review).

### Response of individuals

A total of 19 patients participated in interviews across the four intervention sites. There was a range of responses to the intervention among patients, who provided feedback on the intervention itself and described how they did or did not participate in PUP care. Participants’ perceptions of their experience with the intervention were described in three themes/categories: (1) importance of personal contact in PUPCB delivery; (2) understanding PUP enhances participation; and (3) individual factors impact patients’ engagement in PUP. Patients particularly highlighted the importance of human interaction in facilitating engagement with the PUPCB. An unintended consequence of the study was that study outcome assessors acted as a reminder to patients to enact PUP strategies. Patients responded well to the intervention when they thought it reinforced what they already knew or did. They described how improved knowledge and awareness of PUP empowered and motivated them to participate in PUP care. They also discussed barriers to participation, which included low-perceived importance of PUP and personal factors such as age, cognition and mobility. Further detail on findings from patient interviews are reported elsewhere (citation masked for peer review).

## Discussion

This process evaluation provides important insights into the implementation of a PUPCB and in interpretation of the INTACT trial’s findings. Recruitment and reach was broad, providing a good representation of patients and hospitals; intervention delivery to individuals and clusters was consistently high across sites; and patients and nurses responded positively to the intervention. This shows promise for ease of implementation of a patient-centred PUPCB. The main trial found that intervention patients developed significantly less PU at the cluster level; however, this finding was not significant at the individual patient level after adjusting for covariates and clustering (citation masked for peer review). As this process evaluation showed no major failures relating to implementation of the intervention, this lack of statistical significance at the patient level may be due to the study being underpowered rather than inadequate implementation processes. There are also various other potential reasons for the main study findings, as discussed in the INTACT trial paper [25].

High recruitment and reach of clusters and individuals suggests that both patients and nurses were willing to engage in an intervention encouraging patient participation in PUP care and nurse-patient partnership. This is consistent with two Australian studies that found the majority of patients they interviewed or surveyed wanted to play a proactive role in PUP in hospital [34, 35]. In the context of Australian health care, some of which have incentive or penalty programs in place to reduce PU [13, 16]; it is clearly in hospitals’ best interests to participate in PUP interventions. Patient engagement and PUP are two of the Australian Commission on Safety and Quality in Health Care’s national safety and quality health service standards [13], and are also in line with international PUP clinical practise guidelines [26], which may have contributed to the high participation rate of hospitals in the trial. Due to the broad representation of hospitals and wards, it appears the PUPCB may be practical and feasible in a variety of Australian hospitals. Similarly, the fact that two thirds of the target population (i.e. of eligible patients) participated in the study means that a wide range of patients were represented and were willing to engage with the care bundle. This is consistent with two Australian studies that found the majority of patients they interviewed or surveyed wanted to play a proactive role in PUP in hospital [34, 35]. In retrospect, it would be interesting to better understand why one third of patients declined to participate.

The intervention was implemented and delivered as intended, and all clusters received the intervention (nursing staff training) in a similar way in terms of content, dose and delivery. A range of training delivery methods were used to inform nurses about the PUPCB and instruct them in partnering with patients in PUP care. It is well recognised in theories of adult learning that multiple modes of delivery are important to accommodate for different individuals’ learning styles and preferences [36]. Patients also received information in different visual formats as well as verbal communication. Most patients (97%) received at least one component of the intervention, and 86% received all three components. Interestingly, the most time was spent when patients only received one component of the bundle. Perhaps patients were more engaged or asked more questions when they just had one resource to focus on. Or, perhaps this time, difference reflects the way the individual research assistants (RAs) approached their delivery of the intervention. Given individuals have different learning styles, it is reasonable that patients may not need to receive all components of the intervention for it to have an effect. This is consistent with the concept of minimally disruptive care, which proposes interventions should be prioritised so patients are less burdened and hence more likely to adhere to the important components [37]. Congruently, tailored interventions seek to customise information or strategies to individuals’ characteristics, needs and preferences [38, 39]. Systematic and integrative reviews have found tailored interventions are more likely to be effective and are preferred by participants compared to standard interventions [40, 41]. Future considerations for implementation of the PUPCB may include assessing patients’ learning preferences and tailoring the content or mode of delivery based on this; for example, some patients may prefer a combination of materials, whilst others may rather have a conversation about the content. Also, messages may need to be reinforced with follow-up doses for patients and ongoing support for nurses around the PUPCB.

Nurses and patients generally responded positively to the intervention (citations masked for peer review). Nurses recognised benefits of the PUPCB to both patients and nurses, with improved awareness, communication and participation related to PUP care. Nurses offered suggestions for implementation and maintenance in practise, which included partnering with nurses through communication and dissemination of evidence (both existing evidence and the trial’s findings); leadership and influence (through internal and external facilitators); and keeping the bundle simple (with minimal paperwork and tasks required for PUPCB delivery) (citation masked for peer review). As nurses’ perceptions of an intervention are likely to influence its uptake and use in clinical practise and in turn, its effect [42], their thoughts of the PUPCB were important to explore as part of the INTACT trial’s process evaluation. Nurses’ responses to the PUPCB indicated it may be an acceptable way to implement evidence-based PUP guidelines and engage patients in PUP care.

The patient interview component of the process evaluation found that overall, patients responded positively to the PUPCB and described how learning about PUP empowered them to participate in PUP care (citation masked for peer review). Patients particularly enjoyed interacting with the research assistants (interventionists and outcome assessors). Some patients had trouble remembering information from the education materials, or said they preferred and remembered more from the discussion with the interventionist. This indicates some patients may have benefited from a top-up dose of the intervention to reinforce the messages. Further, outcome assessors acted as a reminder to patients to enact PUP strategies, which was an unintended consequence of the study. However, evaluation was consistent across intervention and control patients, and so, any effect outcome assessors had on the behaviour of intervention patients is likely due to their presence reminding patients of the information they received in the PUPCB. As the care bundle is intended to be delivered by nurses in actual practise, and nurses perform routine skin assessments, this unintended consequence could actually be positive as it mirrors what would likely occur if the PUPCB was adopted into routine practise. That is, nurses’ presence while performing skin assessments may remind patients to enact PUP strategies if they have previously received the PUPCB. Some patients said they preferred a more interactive style of learning, such as through programs on handheld tablets. The role of technology in health care is increasing, and it may be an effective platform by which to engage hospitalised patients in their care (citation masked for peer review). This is an important consideration for future implementation of the PUPCB.

A key strength of the PUPCB was the strong theoretical framework and valid evidence underpinning it. The Medical Research Council (MRC) recommends complex interventions to have strong theoretical foundations and be evidence-based [30]. The PUPCB was informed by the concepts of patient participation in care, which were evident through the following: (1) the focus on building a strong nurse-patient relationship; (2) meaningful exchange of PUP information; (3) training nurses in surrendering some power/control to enable patients to participate in PUP care; and (4) training nurses and patients in mutual/shared decision-making and participation in physical/intellectual care activities [43]. Intervention strategies were also informed by PUP clinical practise guidelines [44] and five systematic reviews on PUP [19–21, 29, 45]. In line with the MRC framework [30], the intervention underwent feasibility testing and piloting to test procedures, estimate recruitment and retention rates, calculate sample sizes and assess patient and nurse acceptability [46, 47]. Patients’ and nurses’ responses to the intervention indicate behaviour change is likely to occur through factors consistent with Rogers’ ‘diffusion of preventative innovations’ theory [48]. Both patients and nurses responded well to the intervention when they found it simple (low complexity), when it aligned with their current knowledge or practises (compatibility) and when they saw benefits to it (relative advantage).

Other process evaluations of cluster-randomised trials using Grant’s framework [32] are of variable rigour and quality. For example, Wallace et al. only assessed one domain of the framework, individuals’ responses to the intervention, using only qualitative methods [49]. Clyne et al. did not clearly describe which domains were evaluated in their process evaluation methodology, and whilst they collected both quantitative and qualitative data, they did not evaluate clusters’ and individuals’ response to the intervention [50]. One protocol paper gave an in-depth description of the intended use of the framework for their process evaluation, covering all evaluation domains and clearly describing methods of assessing each, using a combination of quantitative and qualitative data [51]; however, it appears the study is yet to be conducted. Our study is the first to our knowledge to use Grant et al.’s framework in its entirety (i.e. covering all evaluation domains) for process evaluation of a cluster-randomised trial.

Our study provides important insights into conducting process evaluations for c-RTs using the framework proposed by Grant et al. [32]. Firstly, we recommend process evaluations be conducted a priori, like this one; however we still did not anticipate some of the data that may be needed for further analysis. For example, the study was not powered for exploratory analyses testing for associations between intervention dose/delivery and main trial outcomes, such as development of a PU. If researchers wish to test for these associations, this needs to be considered in the design of the main trial. In our case, increasing the sample size of the main trial for this type of exploratory analyses was not feasible. So, whilst the framework suggests these be conducted, their usefulness and/or feasibility may be limited in some instances. Secondly, a comprehensive process evaluation like this one that covers all domains of the framework is resource intensive. Researchers must consider the budget and time needed for a process evaluation before applying for funding for the main trial. Finally, the role of the process evaluator is important. In this study, the process evaluator started as an independent assessor; but during analysis of process evaluation data, this role expanded to become part of the team evaluating the main trial, due to the important insights gained from the process evaluation in interpreting the main trial’s findings.

## Conclusions

This study explored processes related to the implementation of a PUPCB intervention encouraging active patient participation in PUP care and promoting nurse-patient partnership. As there were no major failures relating to implementation of the intervention, the lack of statistical significance at the patient level in the main trial may be due to the study being underpowered. The PUPCB was informed by theory and evidence around PUP and patient participation. It was easy to deliver (in less than 10 min) and reach a large proportion of the target population. Patients found it easy to understand, but they (and nurses) may have benefited from a top-up dose (reinforcement) of the intervention. Despite this, the PUPCB was found to be acceptable to patients and nurses; therefore, it may be an effective way of empowering patients to participate in their PUP care and promoting evidence-based PUP practise.

## Abbreviations

ANOVA: Analysis of variance
CI: Confidence interval
c-RT: Cluster-randomised trial
HAPU: Hospital acquired pressure ulcer
IQR: Interquartile range
MRC: Medical Research Council
PU: Pressure ulcer
PUP: Pressure ulcer prevention
PUPCB: Pressure ulcer prevention care bundle
